# Histone-related gene WDR77 promotes tumor progression through cell cycle regulation in skin cutaneous melanoma

**DOI:** 10.3389/fimmu.2025.1611112

**Published:** 2025-12-04

**Authors:** Haoxue Zhang, Ke Tang, Yuyao Liu, Shengxiu Liu

**Affiliations:** 1 Department of Dermatovenerology, First Affiliated Hospital of Anhui Medical University, Hefei, Anhui, China; 2 Key Laboratory of Dermatology, Ministry of Education, Hefei, Anhui, China; 3 Inflammation and Immune Mediated Diseases Laboratory of Anhui Province, Anhui Medical University, Hefei, Anhui, China

**Keywords:** melanoma, histone, WDR77, cell cycle, CDC20, prognosis biomarker, therapeutic target

## Abstract

**Background:**

Skin cutaneous melanoma (SKCM) is extremely malignant, leading to poor prognosis. Epigenetic dysregulation, particularly histone modifications, contributes to disease progression. However, effective histone-based prognostic biomarkers are still lacking in clinical practice.

**Methods:**

Transcriptomic data from TCGA-SKCM and five GEO datasets were analyzed. Ten machine learning algorithms were integrated to build 101 prognostic models. The optimal model, based on seven histone-related genes, showed the highest C-index and was validated in both training and validation cohorts. WDR77 was identified as the hub gene by random forest analysis. The expression of WDR77 was profiled in SKCM. Survival analysis, pathway enrichment analyses, single-cell and spatial transcriptomics were performed to investigate the role of WDR77 in SKCM. The therapeutic relevance of WDR77 was also investigated. In vitro experiments were conducted to validate the function of WDR77.

**Results:**

WDR77, a histone methylation factor working with PRMT5, was identified as a key candidate. WDR77 was significantly upregulated in SKCM and correlated with poor clinical outcomes. Pathway analysis showed that WDR77 was primarily associated with cell-cycle dysregulation. CDC20, a cell cycle factor, emerged as a key co-expressed gene. Patients with concurrent high expression of WDR77 and CDC20 had the worst survival outcomes. WDR77 was predominantly expressed in malignant cells across pan-cancer datasets and positively correlated with CDC20. Spatial transcriptomics confirmed their co-localization and co-upregulation in tumor regions. Functional experiments demonstrated that WDR77 promotes proliferation, migration, and cell cycle progression. Overexpression of WDR77 also increased CDC20 protein levels.

**Conclusion:**

WDR77 serves as both a prognostic biomarker and functional regulator in melanoma, highlighting its potential as a therapeutic target.

## Introduction

Skin cutaneous melanoma (SKCM) is the deadliest form of skin malignancies, responsible for the majority of skin cancer-related deaths (1, 2). While surgery can cure most early-stage localized melanoma, metastasis always symbolizes much worse prognosis (3). Recent treatment breakthrough including BRAF/MEK-targeted therapies and immune checkpoint inhibitors (ICIs) have improved survival in advanced melanoma (4–8), but approximately half of patients fail to achieve long-term benefit (8, 9). These ongoing challenges stresses the urgent need for reliable prognostic biomarkers and alternative therapeutic targets to improve prognosis in SKCM.

Histones are the core structural proteins that package DNA into chromatin and play a central role in epigenetic regulation during cancer development (10, 11). Post-translational modifications (PTMs) of histone tails, including acetylation, methylation, phosphorylation, and ubiquitination, form a “histone code” that regulates gene transcription, DNA repair, and cell cycle progression, affecting many cellular processes (12–14). Dysregulated cell cycle is a hallmark of cancer driven by genetic, epigenetic, and environmental factors (15, 16). In melanoma, loss of checkpoint control leads to uncontrolled proliferation, aggressive tumor growth, and treatment resistance, making cell cycle pathways important potential therapeutic targets. Unlike irreversible genetic mutations, epigenetic alterations including histone modifications are pharmacologically reversible and can influence both cell cycle control and immune-related gene expression. For instance, PRMT5, a histone methyltransferase, regulates immune-related gene expression and its inhibition enhances ICI efficacy in melanoma models (17). This supports histone-modifying enzymes as promising therapeutic targets (17, 18). However, few robust prognostic signatures based on HRGs have been developed for SKCM. Moreover, the exact role of many HRGs in melanoma progression has not been systematically analyzed.

To narrow these gaps, we employed an integrative approach that combines systematic machine learning methods for the optimal prognostic signature for SKCM. Then, as an essential cofactor of PRMT5, WDR77 was selected as the most prognostically significant gene, prompting comprehensive study into its role in melanoma pathogenesis. We found that both WDR77 and PRMT5 are upregulated in melanoma. Pathway and correlation analyses revealed that the cell cycle dysregulation was a primary WDR77-associated mechanism and identified CDC20 as a key co-expressed regulator. Through multi-resolution validation including single-cell and spatial transcriptomics, combined with functional experiments, we demonstrated that WDR77 promotes melanoma progression through cell cycle regulation. These findings establish WDR77 as both a prognostic biomarker and potential therapeutic target. Since PRMT5 inhibitors are already being tested in clinical trials, our work raises the possibility of combining them with immunotherapy in patients with limited treatment options.

## Materials and methods

### Data acquisition and processing

Bulk RNA-sequencing data (HTSeq-FPKM format) and corresponding clinical information for SKCM patients were obtained from the public platforms, The Cancer Genome Atlas (TCGA) (https://portal.gdc.cancer.gov/) and Gene Expression Omnibus (GEO) (https://www.ncbi.nlm.nih.gov/geo/). One TCGA-SKCM dataset and five GEO datasets (GSE19234, GSE22153, GSE54467, GSE65904, and GSE91061) were included. The following inclusion criteria were set: (i) pathologically confirmed SKCM diagnosis; (ii) available overall survival (OS) data with OS>30 days and documented survival status; (iii) available gene expression data without excessive missing values. The TCGA-SKCM dataset comprised 446 samples. GSE19234, GSE22153, GSE54467, GSE65904, and GSE91061 datasets contained 38, 54, 71, 210 and 51 samples, respectively. Normal skin tissue transcriptomic data were retrieved from the Genotype-Tissue Expression (GTEx) project to establish baseline expression profiles for differential expression analysis. To minimize inter-batch effects, the “ComBat” function from the “sva” R package (19, 20) was applied to harmonize gene expression profiles and reduce biological heterogeneity (21). GEO datasets were then merged into a unified GEO cohort and all datasets were subsequently integrated into a Meta cohort for comprehensive analysis. **Supplementary Data 1** summarizes the detailed baseline characteristics of the TCGA samples.

### Identification of histone-related genes and prognostic HRGs

Histone-related genes (HRGs) were identified using the Gene Ontology (GO) knowledgebase (http://geneontology.org/), a comprehensive repository of gene functional annotations (22). Using the search term “histone” and filter criteria “homo-sapiens” and “protein”, all genes associated with histone biology were retrieved (**Supplementary Data 2**). These genes were consistently detected across all six datasets (TCGA-SKCM and five GEO cohorts) and subjected to further analysis.

Univariate Cox proportional hazards regression analysis was performed to identify prognostic HRGs associated with OS in the TCGA-SKCM cohort. Genes with P<0.01 were considered statistically significant and retained for subsequent model construction.

### Machine learning-based construction of histone-related signature

To construct a robust prognostic signature, ten machine learning algorithms were utilized, each with distinct advantages: regularization-based methods (Lasso, Ridge, Elastic Net (Enet)) for variable selection and handling multicollinearity; tree-based methods (Random Survival Forest (RSF)) for capturing non-linear relationships; boosting methods (CoxBoost, Generalized Boosted Regression Modeling (GBM)) for sequential error correction; dimension reduction methods (partial least squares regression for Cox (plsRcox), Supervised Principal Components (SuperPC)) for feature extraction; and classical methods (Stepwise Cox, Survival Support Vector Machine (survival-SVM)) for baseline comparison.

Using the prognostic HRGs from the previous step, 101 prediction models were constructed based on both single-algorithm approaches and pairwise algorithm combinations with different parameters, which were evaluated in the TCGA-SKCM cohort within a leave-one-out cross-validation (LOOCV) framework. The LOOCV framework is selected to maximize the use of training data and provide almost unbiased performance evaluation results. Subsequently, each model was validated in five external validation datasets (GSE19234, GSE22153, GSE54467, GSE65904, and GSE91061). The Harrell’s concordance index (C-index), a standard metric quantifying the discriminative ability of survival models by assessing the concordance between predicted risk and observed survival times, was calculated for each model. The model with the highest average C-index was selected as the optimal consensus HRS.

For each patient, the risk score was calculated according to the expression levels of signature genes weighted by their corresponding coefficients derived from the optimal model. In each dataset, patients were divided into high-risk and low-risk groups using the median risk score as the cut-off value.

### Validation of HRS prognostic performance

Kaplan-Meier (KM) survival analysis with log-rank test was adopted to compare survival outcomes between high-risk and low-risk groups in the Meta cohort, GEO cohort, and six independent datasets. Univariate Cox survival analysis was conducted to evaluate the prognostic value of HRS in each cohort, and the results were synthesized through meta-analysis with forest plots to assess the consistency of HRS performance across different cohorts and estimate the pooled hazard ratio.

### Hub gene identification and differential expression analysis

To identify the most important prognostic gene within HRS, random forest analysis was performed using the “randomForestSRC” R package. Variable importance was calculated for all seven signature genes (WDR77, PARP9, KPNA2, GATAD2A, GATA3, ARID5A, and IKZF3) based on their contribution to prediction accuracy, and genes were ranked accordingly. Consequently, WDR77 was selected as the hub gene with the highest variable importance score for subsequent in-depth validation.

Differential expression analysis of WDR77 between normal skin tissues and melanoma samples using the integrated TCGA-SKCM-GTEx data. The Wilcoxon rank sum test was applied to evaluate the statistical significance. In order to explore the relationship between WDR77 expression and somatic mutations, the “independence_test” function in the “coin” R package was used for an arrangement-based independence test. This non-parametric method generates an arrangement distribution by randomly remarking the original data, so as to evaluate the independence between the gene expression level and the mutation state. Only genes with a mutation frequency greater than 10% in the TCGA-SKCM cohort were included in the analysis, and genes showing significant correlation (P<0.01) were retained for visualization.

At the protein level, immunohistochemistry (IHC) images from the Human Protein Atlas (HPA) database (https://www.proteinatlas.org) (23) were retrieved to validate WDR77 protein expression between normal skin and melanoma tissues. Three independent anti-WDR77 antibodies (HPA026437, HPA026448, and HPA027271) were used for comparative evaluation. For detailed patient information of all IHC samples, including demographics and pathological diagnoses, please refer to the **Supplementary Data 3**.

To validate the specificity of WDR77’s role in melanoma, its expression was further examined in other dermatoses. For cutaneous squamous cell carcinoma (cSCC), spatial transcriptomics data from three independent hematoxylin and eosin (HE)-stained tissue sections (GSE144239-GSM4565825-P6-rep1 (24), V10F24-015-A1 (25), and V10F24-015-B1 (25)) were analyzed by comparing malignant, boundary, and non-malignant regions, while for psoriasis and atopic dermatitis (AD), bulk RNA-sequencing data from multiple cohorts were analyzed by comparing lesional versus non-lesional samples. The Wilcoxon rank-sum test was applied to assess statistical significance. Dataset information is provided in **Supplementary Data 4**.

### Subcellular localization, causal inference, and clinical significance assessment of WDR77

The subcellular localization of WDR77 was first assessed using immunofluorescence images from the HPA database. These images were obtained from established cell lines (A-431, U-251MG, and U2OS) stained with the HPA027271 antibody. For experimental validation, immunofluorescence staining was performed in Mewo melanoma cells following standard protocols.

To explore the potential causal relationship between WDR77 expression and SKCM development, Mendel randomization followed by expression quantitative trait loci (eQTL) and genome-wide association study (GWAS) colocalization analysis was performed.

For prognostic evaluation, TCGA-SKCM samples were stratified into quartiles according to WDR77 expression levels (Q1: top 25%; Q2: 25-50%; Q3: 50-75%; Q4: bottom 25%). KM analysis was conducted to compare disease-specific survival (DSS), overall survival (OS), and progression-free Interval (PFI) among the four groups, and the log-rank test was used to assess the statistical significance. The chi-square test was applied to compare differences in survival status (alive vs. dead) quartiles.

Furthermore, to investigate the relationship with metastasis, WDR77 expression levels across N staging groups in the TCGA-SKCM cohort were compared using the Wilcoxon rank-sum test for specific pairwise comparisons between each early-stage group (N0, N1, N2) and the N3 group, and the chi-square test was used to assess the distribution of N stages between high- and low-WDR77 expression groups.

In addition, a meta-analysis integrating Univariate Cox regression results from multiple GEO and TCGA datasets was implemented to validate the consistency of prognostic associations across independent cohorts. The standard error of the hazard ratio (HR) was calculated using the 95% confidence interval (CI).

### Comprehensive mechanistic exploration of WDR77

To investigate the association between WDR77 expression and cancer-related functional states in melanoma, the Z-score algorithm (26) was applied to normalize the expression of WDR77 and gene set variation analysis (GSVA) scores of 14 functional states (stemness, invasion, metastasis, proliferation, EMT, angiogenesis, apoptosis, cell cycle, differentiation, DNA damage, DNA repair, hypoxia, inflammation, and quiescence) derived from CancerSEA (http://biocc.hrbmu.edu.cn/CancerSEA/) (27). Pearson correlation analysis was then conducted between WDR77 expression Z-score and the 14 functional states’ Z-scores.

To explore differentially enriched pathways, the samples with the top 30% highest WDR77 expression levels were defined as the high-expression group, while those with the bottom were defined as the low-expression group. Kyoto encyclopedia of genes and genomes (KEGG) enrichment analysis was conducted using the “clusterProfiler” R package to identify differentially enriched signaling pathways in the high-expression group. The “limma” R package was used to conduct the differential analysis, and the logF2 values were calculated as indicators to rank all genes. For gene set enrichment analysis (GSEA), differential analysis was first conducted using the “limma” R package, and the resulting log2 fold changes were used to rank all genes. This ranked gene list was then subjected to GSEA using the “clusterProfiler” R package. Enrichment scores (ES) were calculated with statistical significance assessed through permutation testing.

To evaluate the functional necessity of WDR77 in cancer cell survival, the Cancer Dependency Map (DepMap) database (https://depmap.org/portal/) which provides dependency scores based on CRISPR-Cas9 knockout screens across pan-cancer cell lines, was analyzed (28). Dependency scores quantify gene essentiality, with negative scores meaning cell growth inhibition and/or cell death after gene knockout.

Given the established functional partnership between WDR77 and PRMT5, the expression pattern of PRMT5 in the TCGA-SKCM-GTEx dataset was validated, and the association between PRMT5 and WDR77 expression was assessed by comparing WDR77 levels across PRMT5 expression quartiles using the Kruskal-Wallis test. To further investigate novel molecular mechanisms underlying the association between WDR77 expression and cell-cycle regulation, PARADIGM pathways analysis was performed to identify pathway components closely correlated with WDR77. Subsequently, both WDR77 and CDC20 expression were standardized using Z-score, with Z-score ≤ 0 defined as low expression and Z-score > 0 as high expression, thereby generating four subgroups: WDR77-/CDC20-, WDR77-/CDC20+, WDR77+/CDC20-, and WDR77+/CDC20+. KM survival analysis was conducted using the “survival” R package, and the “survfit” function was implemented for the log-rank test to evaluate survival differences among the four subgroups.

### Single-cell and spatial transcriptomics analysis

Single-cell expression data files were downloaded from the Tumor Immune Single-cell Hub 2 (TISCH2) (http://tisch.compbio.cn/home/) database (29) for pan-cancer analysis. The “pheatmap” R package was used to establish a heatmap displaying the WDR77 landscape in pan-cancer. For hierarchical clustering, Euclidean distance and Ward’s minimum variance method were used.

For SKCM-specific analysis, two single-cell datasets (SKCM_GSE115978_aPD1 (30) and SKCM_GSE72056 (31)) were analyzed. Uniform Manifold Approximation and Projection (UMAP) (32) was applied for dimensionality reduction and visualization of cell clusters. The Kruskal-Wallis Rank Sum Test was used to compare WDR77 expression levels among different cell types.

To assess the co-expression patterns between WDR77 and CDC20, Spearman correlation analysis was conducted on 189 pan-cancer single-cell datasets. Their relationship in malignant cells from two SKCM single-cell datasets was also analyzed.

To complement single-cell findings with spatial context, spatial transcriptomics data (33) generated using the 10× Genomics (https://www.10xgenomics.com/cn) Visium platform were analyzed. Cell type deconvolution was applied to assess the cell composition of each spot using the “CottraZm” R package (34) and the SpatialTME database (https://www.spatialtme.yelab.site/) (35), with integrated data accessed via the Sparkle database (https://www.grswsci.top). WDR77 and cell type scores were visualized using the “SpatialFeaturePlot” function in the “Seurat” R package, and the higher the score, the darker the color, the higher the amount of this cell type in the spot.

Spots were classified into three groups based on malignant cell scores: Malignant (score = 1), Normal (score = 0), and Mixed (0 < score < 1). The Wilcoxon rank sum test was used to compare WDR77 and CDC20 expression among the three groups. Spearman correlation analysis was performed to evaluate correlations between cell type composition and gene expression, and the “linkET” R package was applied for visualization.

### Immune infiltration, genomic stability, and epigenetic analysis

To investigate the relationship between WDR77 expression and tumor immune microenvironment, immune cell infiltration levels were quantified estimated using multiple computational algorithms implemented in the TIMER2.0 database (https://compbio.cn/timer2/) (36). Spearman correlation analysis was performed to understand the relationship between WDR77 expression and different cell types and WDR77 expression. Besides, Spearman correlation analysis was performed between WDR77 expression with both the infiltration levels of various immune cell types and the Tracking Tumor Immunophenotype (TIP) (http://biocc.hrbmu.edu.cn/TIP/) scores (37). The correlation matrix involving TIP scores was visualized using the “linkET” R package.

For microsatellite instability (MSI) analysis, WDR77 levels were compared across MSI-high (MSI-H), MSI-low (MSI-L), and microsatellite stable (MSS) subtypes in colon adenocarcinoma (COAD) and stomach adenocarcinoma (STAD) cohorts. The chi-square test was employed to display patient distribution across WDR77 high/low groups and MSI subtypes.

To examine the epigenetic regulation of WDR77, its promoter methylation data (Illumina 450K array) was obtained from the TCGA-SKCM cohort. Samples were stratified into hypermethylation and hypomethylation groups based on median promoter methylation levels, and DSS was compared using KM survival analysis with the log-rank test.

### Cell culture and transfection

Four melanoma cell lines, Mewo (Cellverse, Shanghai, China), M14 (Immocell, Xiamen, China), A375, and WM115 (Pricella, Wuhan, China), were cultured in DMEM (MeilunBio, Dalian, China) supplemented with 10% fetal bovine serum (FBS; MeilunBio, Dalian, China) and 1% antibiotics at standard culture conditions (37°C, 5% CO2). Cell line authenticity was validated by short tandem repeats (STR) profiling.

For functional studies, WDR77 was manipulated through overexpression and CRISPR/Cas9-mediated knockout. For overexpression, cells were transfected with a plasmid encoding full-length human WDR77 (pLV3-CMV vector) or an empty vector control. For knockout, cells were transfected with the LentiCRISPRv2 vector (38) plasmid containing a specific sgRNA targeting WDR77 or a non-targeting sgRNA control. All transfections were performed using the jetOPTIMUS^®^ DNA Transfection Reagent (Polyplus, FRANCE) according to the manufacturer’s instructions. Cells were harvested 72 hours post-transfection for transfection efficiency validation and subsequent functional assays.

### Quantitative real-time PCR

Total RNA was extracted from cells using the MiPure Cell miRNA Kit (Vazyme, Nanjing, China), and RNA concentration was determined using a NanoDrop spectrophotometer. RNA was reverse transcribed into cDNA using the PrimeScript™ RT Reagent Kit (Takara, Beijing, China). qRT-PCR reaction was conducted using an Applied Biosystems thermal cycler (ThermoFisher, USA). Primers sequences were as follows: WDR77 forward, CTGGCTTTTTAAGGACCCCTG; reverse, TCTCCCCAACCCAAGTGAGG; GAPDH forward, AAGGTGAAGGTCGGAGTCAA; reverse, AATGAAGGGGTCATTGATGG.

### Western blot analysis

Total protein was extracted from cells using RIPA lysis buffer supplemented with protease and phosphatase inhibitors. Protein concentration was determined using the BCA assay. Equal amounts of protein were separated by SDS-PAGE and transferred to PVDF membranes. Membranes were blocked, incubated overnight at 4°C with primary antibodies against WDR77 (Abcam, UK), CDC20 (Proteintech, Wuhan, China) and GAPDH (Proteintech, Wuhan, China), followed by HRP-conjugated secondary antibodies. Protein bands were visualized using enhanced chemiluminescence (ECL).

### Immunofluorescence staining

Mewo cells were cultured on glass coverslips and fixed with 4% paraformaldehyde (PFA) in PBS for 15 minutes at room temperature. After permeabilization with 0.5% Triton X-100 (Beyotime, Shanghai, China) for 15 minutes, cells were blocked with 5% bovine serum albumin (BSA) for 30 minutes. Cells were then incubated with anti-WDR77 primary antibody (Abcam, UK) overnight at 4°C, followed by fluorescence-conjugated secondary antibody for 1 hour at room temperature. Nuclei were counterstained with DAPI, and images were captured using a fluorescence microscope.

### Cell proliferation assays

Cell viability was assessed using the Cell Counting Kit-8 (CCK-8) (TargetMol, USA) according to the manufacturer’s protocol. Cells were seeded in 96-well plates at a density of 3,000 cells/well. At the specified time point (0, 24, 48, and 72 hours), CCK-8 reagent was added to each well and incubated at 37°C for 1 hour. The absorbance at 450 nm was measured using a microplate reader. The 5-ethynyl-2′-deoxyuridine (EdU) incorporation assay was performed using the BeyoClick™ EdU Cell Proliferation Kit with Alexa Fluor 488 (Beyotime, Shanghai, China). Transfected cells were seeded in 6-well plates at 1×10^6^ cells/well and cultured overnight. The cells were incubated with EdU for 4 hours, then fixed and permeable, and stained with Alex fluorescent 488 activator and Hoechst 33342 according to the manufacturer’s instructions. Capture images of three random areas under a fluorescence microscope. The percentage of EdU-positive cells is calculated as follows: EdU-positive rate = (EdU-positive cell count/total cell count) × 100%.

### Cell migration assays

Cells were seeded in 6-well plates and cultured to approximately 80% confluence. Scratch wounds were created using sterile 100μL pipette tips. Cells were washed three times with PBS, and serum-free medium was added to minimize proliferation effects during the 48-hour observation period. Images were captured immediately after scratching (0 h) and after a 48-hour incubation period using an inverted microscope. Cell migration rate was calculated using ImageJ software as follows: Migration Rate = [(Initial Scratch Area - Final Scratch Area)/Initial Scratch Area] × 100%. Cell migration was also assessed using Transwell chambers (8 μm pore size, 12-well format). Cells were seeded in the upper chamber at a density of 5×10^4^ cells in 500 μL serum-free medium, while the lower chamber contained 1.5 mL complete medium with 10% FBS as a chemoattractant. After 24 hours, non-migrated cells on the upper surface were removed using PBS-soaked cotton swabs. Migrated cells on the lower membrane surface were fixed with 4% paraformaldehyde for 20 minutes, stained with 0.5% crystal violet, and counted under a microscope.

### Flow cytometry assays

For apoptosis analysis, cells were stained using the Annexin V-Alexa Fluor 647/PI Apoptosis Detection Kit (Yeasen, Shanghai, China) according to the manufacturer’s protocol. Stained cells were kept on ice and analyzed by flow cytometer within 1 hour. For cell cycle analysis, cells were collected 48 hours post-transfection and fixed in 70% ethanol at −20°C overnight. Fixed cells were washed with PBS, resuspended in 0.5 mL propidium iodide (PI) staining solution containing RNase A (Cell Cycle Analysis Kit, Beyotime, Shanghai, China), and incubated at 37°C in the dark for 30 minutes. DNA content was analyzed using a BD LSRFortessa flow cytometer (BD Biosciences, USA), and the percentages of cells in G1, S, and G2/M phases were quantified using FlowJo software.

### Statistical analysis

All bioinformatic analyses were performed using R software (version 4.3.3). Gene expression levels were standardized to z-scores using the scale function. KM survival analysis and univariate Cox regression were conducted using “survival” and “survminer” R packages to evaluate DSS, PFI, and OS. Hazard ratios (HRs) with 95% confidence intervals (CIs) were calculated. The log-rank test was used to assess survival differences. The Wilcoxon rank-sum test was used for two-group comparisons, and the Kruskal-Wallis test for multiple-group comparisons. Pearson correlation was applied for normally distributed data, while Spearman correlation was used for non-normally distributed data. The chi-square test was performed for categorical variables. Pathway activity was evaluated using the “GSVA” package, and Gene Set Enrichment Analysis (GSEA) was performed using the “clusterProfiler” package. Methylation data were annotated using the “ChAMPdata” package. Experimental data were derived from at least three independent replicates. A two-sided P-value of less than 0.05 was considered statistically significant.

## Results

### Integration of various machine learning approaches identifies the optimal histone-related signature for SKCM prognosis

**Figure 1:**
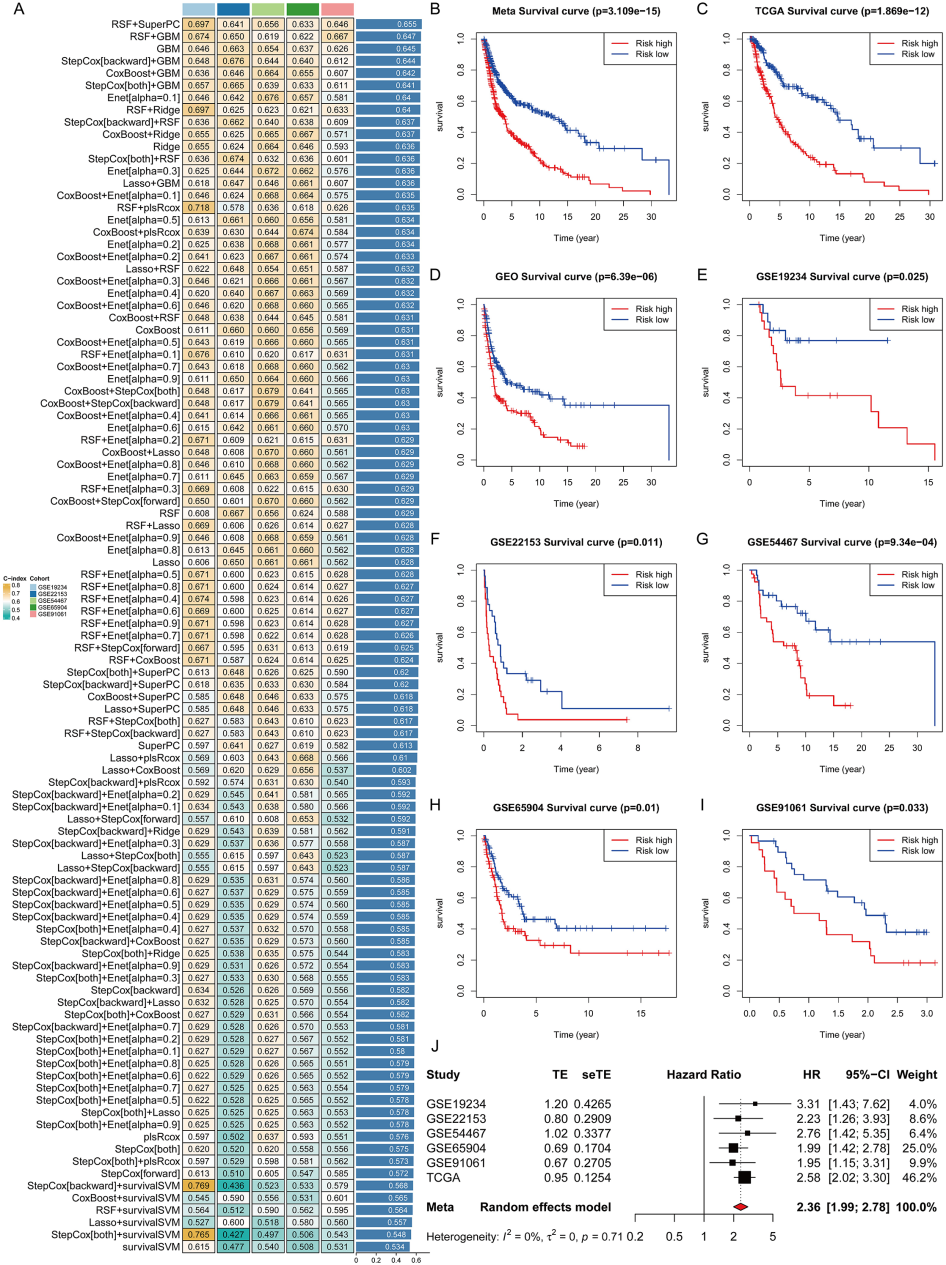
Development and validation of a consensus histone-related signature (HRS) via machine learning-based integrative approaches. **(A)** 101 prediction models based on 10 machine learning algorithms, developed using the LOOCV framework, with C-indexes calculated across all validation datasets. **(B-I)** Kaplan–Meier (KM) survival curves stratified by HRS in meta-cohort (P = 3.109e−15) **(B)**, TCGA-SKCM (P = 1.869e−12) **(C)**, GEO cohort (P = 6.39e−06) **(D)**, GSE19234 (P = 0.025) **(E)**, GSE22153 (P = 0.011) **(F)**, GSE54467 (P = 9.34e−04) **(G)**, GSE65904 (P = 0.01) **(H)**, GSE91061 (P = 0.033) **(I)**. **(J)** Meta forest map validating the prognostic performance of HRS across independent validation datasets.

Univariate Cox regression identified 89 prognostic histone-related genes (HRGs) (P < 0.01) from 866 HRGs. Among 101 algorithm combinations evaluated, the combination of RSF and SuperPC achieved the highest average C-index (0.655) and was selected as the optimal histone-related signature (HRS) (**Figure 1A**). The HRS demonstrated satisfying prognostic stratification capability in the Meta cohort, GEO cohort, and all six independent datasets (TCGA-SKCM, GSE19234, GSE22153, GSE54467, GSE65904, and GSE91061). High-risk group patients consistently exhibited significantly adverse survival outcomes compared to low-risk patients (all P < 0.05; **Figures 1B–I**). Similar prognostic performance was observed in the integrated GEO cohort and Meta cohort. Meta-analysis (**Figure 1J**) further confirmed the robust and persistent prognostic value of HRS across all independent validation datasets.

### WDR77 is the hub HRG with elevated expression in melanoma

**Figure 2:**
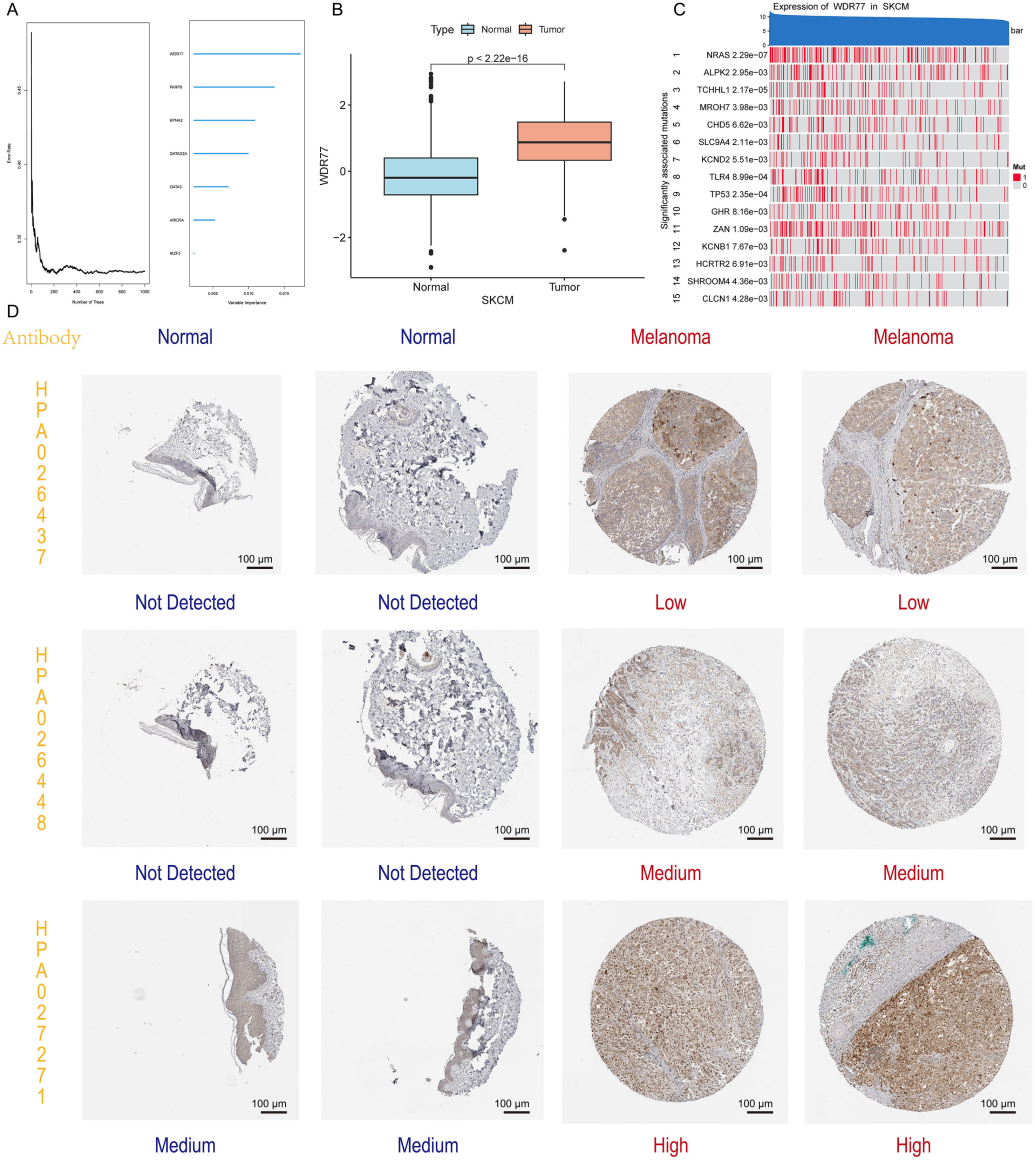
Identification of WDR77 as the hub gene and validation of its differential expression. **(A)** Random forest variable importance analysis identifying WDR77 as the most critical prognostic gene among seven signature genes. **(B)** Box plot showing significantly elevated WDR77 expression between normal tissues and melanoma tissues using integrated TCGA-GTEx data (P < 2.22e-16). **(C)** Heatmap profiling mutation-related genes significantly correlated with WDR77 expression. Only genes with mutation frequency >10% and P < 0.01 are shown. **(D)** Immunohistochemistry images from the HPA database demonstrating WDR77 protein expression in normal skin and melanoma tissues using three antibodies (HPA026437, HPA026448, and HPA027271). Scale bar: 100 μm.

Random forest analysis identified WDR77 as the most important prognostic gene among seven signature genes (WDR77, PARP9, KPNA2, GATAD2A, GATA3, ARID5A, and IKZF3), because its variable importance score was the highest (**Figure 2A**). Compared with normal skin tissues, the expression of WDR77 in melanoma tissues increased significantly (P < 0.05, **Figure 2B**). Furthermore, its expression was closely related to mutations and was associated with key driving genes such as NRAS, ALPK2, and TP53 (**Figure 2C**). At the protein level, immunohistochemistry using three different antibodies (HPA026437, HPA026448, and HPA027271) consistently demonstrated elevated WDR77 expression in melanoma compared to normal skin (**Figure 2D**). Besides, WDR77 was found to be upregulated in cutaneous squamous cell carcinoma, atopic dermatitis, and psoriasis compared to non-lesional skin (**Supplementary Figures 1A–E**).

### WDR77 exhibits diverse intracellular localizations and is associated with poor prognosis in SKCM

**Figure 3:**
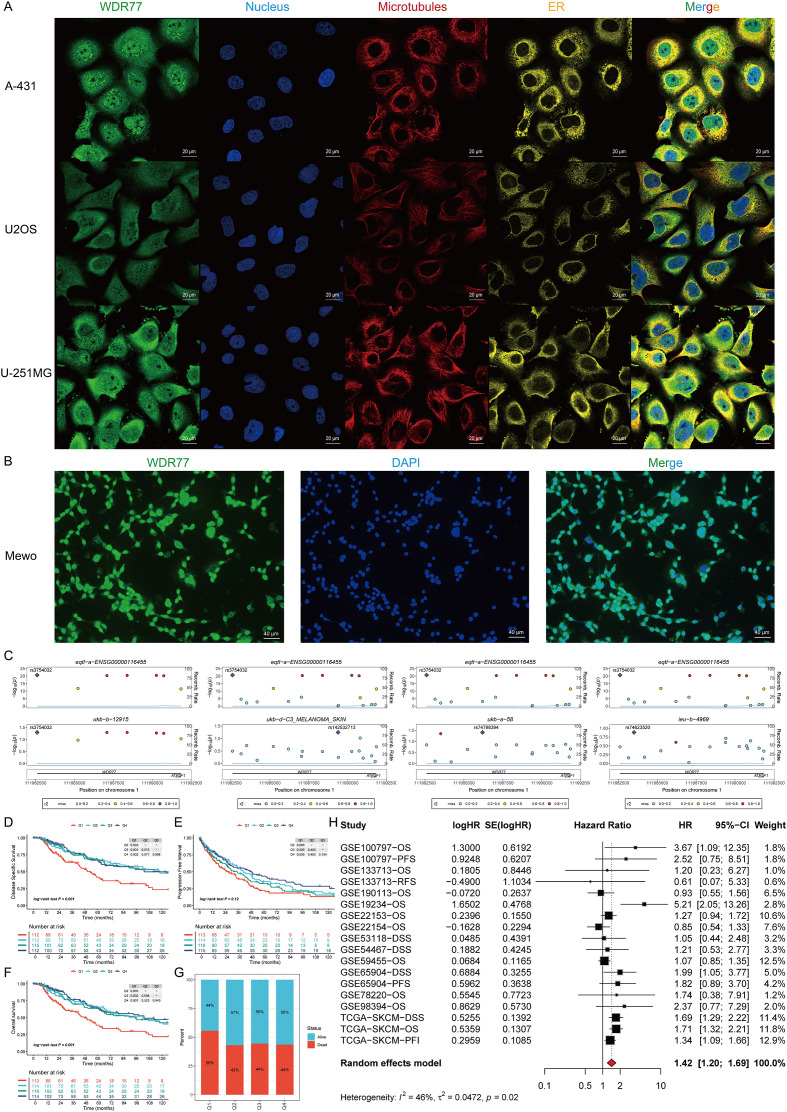
Subcellular localization and clinical significance of WDR77 in SKCM. **(A)** Immunofluorescence images from the HPA database showing WDR77’s wide expression across different cellular compartments. Scale bar: 20 μm. **(B)** Immunofluorescence of WDR77 localization in Mewo cells. WDR77 (green) exhibits nuclear distribution with co-localization with DAPI (blue). Scale bar: 40 μm. **(C)** Mendelian randomization eQTL-GWAS colocalization analysis showing no shared causal variants between WDR77 and SKCM across multiple GWAS datasets. **(D-F)** KM curves showing that patients in the Q1 group (highest WDR77 expression quartile) exhibit the worst outcomes according to DSS **(D)**, PFI **(E)**, and OS **(F)**. **(G)** Bar plot showing the highest mortality rate in the Q1 group. **(H)** Forest plot from meta-analysis demonstrating the adverse role of high WDR77 expression across multiple independent datasets.

Immunofluorescence imaging revealed that WDR77 is mainly located in nucleoplasm and cytosol, but also in the Golgi body (**Figure 3A**). Experimental verification in Mewo melanoma cells confirmed the nuclear localization of WDR77 (**Figure 3B**). Mendel randomized eQTL-GWAS colocalization analysis showed that WDR77 and SKCM do not share causal variants in multiple GWAS-SKCM datasets (**Figure 3C**), indicating an indirect impact on the progression of SKCM.

KM curves showed that there are significant differences between the four groups (Q1-Q4) in DSS, PFI, and OS (**Figures 3D–F**). Patients with the highest expression of WDR77 in the Q1 group had the shortest survival. The mortality rate of the Q1 group was also the highest (56%), which was significantly higher than the lower-expressed quartiles (**Figure 3G**). Consistent with these findings, a meta-analysis across multiple independent cohorts showed that elevated WDR77 expression results in adverse survival (HR > 1, P < 0.05), despite obvious heterogeneity across datasets (**Figure 3H**).

### WDR77 expression is associated with cell-cycle pathways and correlates with CDC20 in SKCM

**Figure 4:**
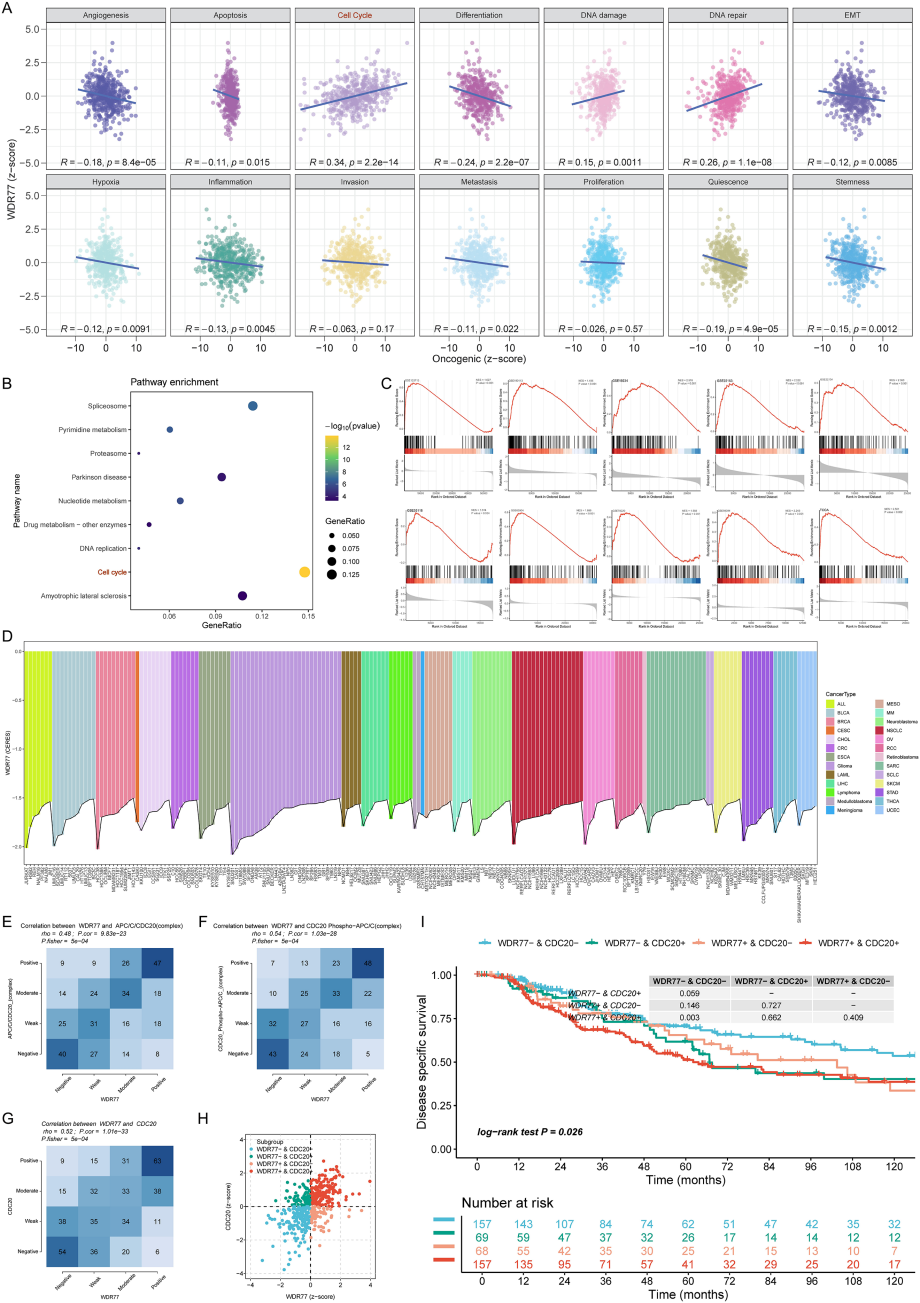
Pathway enrichment analyses reveal WDR77’s association with cell cycle regulation and CDC20. **(A)** Heatmap showing Pearson correlations between WDR77 and 14 cancer-related functional states. **(B)** KEGG enrichment analysis showing cell-cycle pathway enrichment in the WDR77-high expression group. **(C)** GSEA results demonstrating cell-cycle related gene enrichment in the WDR77-high expression group. **(D)** CRISPR-Cas9 data demonstrating WDR77 knockout leads to cell growth arrest and/or cell death in pan-cancer cell lines. **(E–G)** PARADIGM pathways analysis revealing positive correlations between WDR77 and APC/C/CDC20_(complex) (r = 0.48) **(E)**, CDC20_Phospho-APC/C_(complex) (r = 0.54) **(F)**, and CDC20 (r = 0.52) **(G)**, all P < 0.05. **(H)** Scatter plot showing patient stratification into four subgroups based on WDR77/CDC20 expression status, with red dots representing the WDR77+/CDC20+ group. **(I)** KM curves comparing DSS across the four subgroups, demonstrating the worst prognosis in WDR77+/CDC20+ patients.

WDR77 expression was positively correlated with the cell-cycle pathway in SKCM (**Figure 4A**). In line with this, KEGG enrichment analysis verified that the WDR77-high expression group is mainly enriched in the cell-cycle signaling pathway (**Figure 4B**). GSEA results across TCGA and nine GEO datasets further confirmed significant enrichment of cell-cycle-related genes in the WDR77-high expression group (**Figure 4C**). Collectively, these results implicate WDR77 in the regulation of the cell cycle. Across pan-cancer cell lines, WDR77 knockout resulted in cell growth arrest or apoptosis (**Figure 4D**).

PRMT5 was significantly upregulated in melanoma tissues compared to normal skin (P < 0.001, **Supplementary Figure 2A**). In the Q1 group with the highest PRMT5 expression, the WDR77 expression level was also the highest (P = 0.02), confirming their positive associations (**Supplementary Figure 2B**). PARADIGM pathways analysis showed positive correlations between WDR77 expression and three cell-cycle regulators: APC/C/CDC20_(complex), CDC20_Phospho-APC/C_(complex), and CDC20 (r = 0.48, 0.54, 0.52, respectively; all P < 0.05) (**Figures 4E–G**). To assess the interaction between WDR77 and CDC20, patients were divided into four groups: WDR77-/CDC20-, WDR77-/CDC20+, WDR77+/CDC20-, and WDR77+/CDC20+, and visualized using different colored dots with red representing (co-high expression of both genes) (**Figure 4H**). KM analysis proved that patients in the WDR77-/CDC20- group exhibit significantly better DSS compared to other groups. Notably, co-expression of WDR77 and CDC20 led to the poorest DSS in SKCM (P = 0.026) (**Figure 4I**).

### WDR77 is primarily expressed in malignant cells at single-cell and spatial resolution

**Figure 5:**
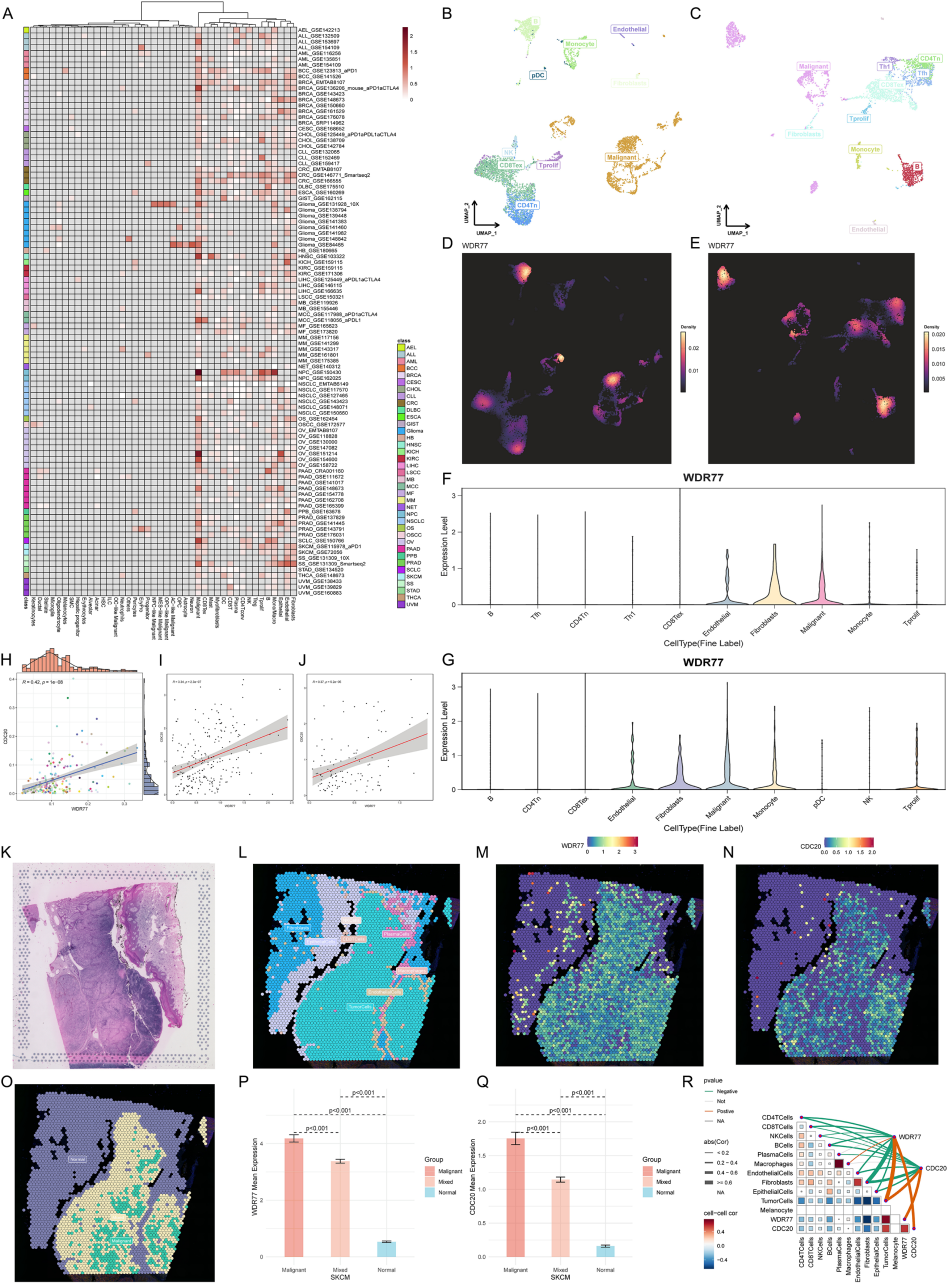
Single-cell and spatial transcriptome analyses reveal WDR77 enrichment in malignant cells and positive correlations with CDC20. **(A)** Pan-cancer heatmap showing WDR77 enrichment in malignant cells across multiple tumor types. **(B–E)** UMAP visualizations displaying cell clusters **(B, C)** and WDR77 expression density **(D, E)** in two SKCM single-cell datasets: SKCM_GSE115978_aPD1 and SKCM_GSE72056. Brighter dots indicate higher WDR77 expression. **(F, G)** Violin plots confirming significantly elevated WDR77 expression in malignant cells compared to other cell types in both datasets. **(H–J)** Scatter plots showing positive correlations between WDR77 and CDC20 across 189 pan-cancer single-cell datasets **(H)**, and within WDR77+/CDC20+ malignant cells from SKCM single-cell datasets **(I, J)**. **(K–O)** Spatial transcriptomics of melanoma tissue. Each spot (diameter ~55 μm) is a tissue microregion colored by cell type composition or gene expression level. The panels show H&E staining **(K)**, malignant cell distribution **(L)**, WDR77 expression **(M)**, CDC20 expression **(N)**, and their spatial overlap **(O)**. **(P, Q)** Bar charts show WDR77 and CDC20 are expressed at much higher levels in malignant regions than in mixed malignant and normal regions (P < 0.001). **(R)** Correlation matrix showing relationships between tumor cells and WDR77, tumor cells and CDC20, and WDR77 and CDC20. Line color shows correlation direction (red: positive; green: negative; grey: non-significant), and line thickness shows correlation strength. In the triangular matrix, square size shows correlation strength and color intensity shows statistical significance (darker colors = lower P values).

In pan-cancer, WDR77 was preferentially highly expressed in malignant cells (**Figure 5A**). In two SKCM-specific single-cell datasets (SKCM_GSE115978_aPD1 and SKCM_GSE72056), UMAP visualization identified distinct cell clusters including malignant cells, B cells, and other cell types (**Figures 5B, C**). WDR77 expression density plots further illustrated this intensive localization in malignant cells, with higher expression intensity represented by brighter dots (**Figures 5D, E**). Violin plots quantified that WDR77 expression is significantly elevated in malignant cells compared to other cell types in both datasets (**Figures 5F, G**). Next, Spearman correlation analysis across 189 pan-cancer single-cell datasets revealed a positive correlation between WDR77 and CDC20 (r = 0.42, P < 0.001) (**Figure 5H**). Then, this association was validated in WDR77+/CDC20+ malignant cells from the two SKCM datasets (r = 0.34 and 0.37, respectively; P < 0.001) (**Figures 5I, J**).

Spatial transcriptomics analysis corroborated the above observations. Deconvolution-based clustering of 10× Genomics Visium data demonstrated spatial overlap among malignant cells, mixed malignant cells, WDR77 expression, and CDC20 expression (**Figures 5K–O**). Both WDR77 and CDC20 exhibited significantly higher expression in malignant regions compared to mixed malignant and normal regions (both P < 0.001) (**Figures 5P–Q**). Close correlations were observed between WDR77 and CDC20, WDR77 and tumor cells, and CDC20 and tumor cells (**Figure 5R**).

### WDR77 has strong associations with tumor immunity and genomic stability

We found that WDR77 expression was correlated with immune cell infiltration levels in melanoma estimated by multiple computational algorithms (**Supplementary Figure 3**). Also, WDR77 expression showed negative associations with CD8+ T cell recruitment in melanoma (**Supplementary Figure 4A**). Moreover, evaluation of microsatellite instability (MSI) subtypes in COAD and STAD cohorts showed significantly lower WDR77 expression in MSI-high tumors compared to MSI-low and microsatellite stable subtypes (**Supplementary Figures 4B–E**). WDR77 promoter hypermethylation correlated with improved DSS in SKCM patients (P = 0.026, **Supplementary Figure 4F**).

### WDR77 promotes the proliferation and migration of melanoma cells and regulates the cell cycle process

**Figure 6:**
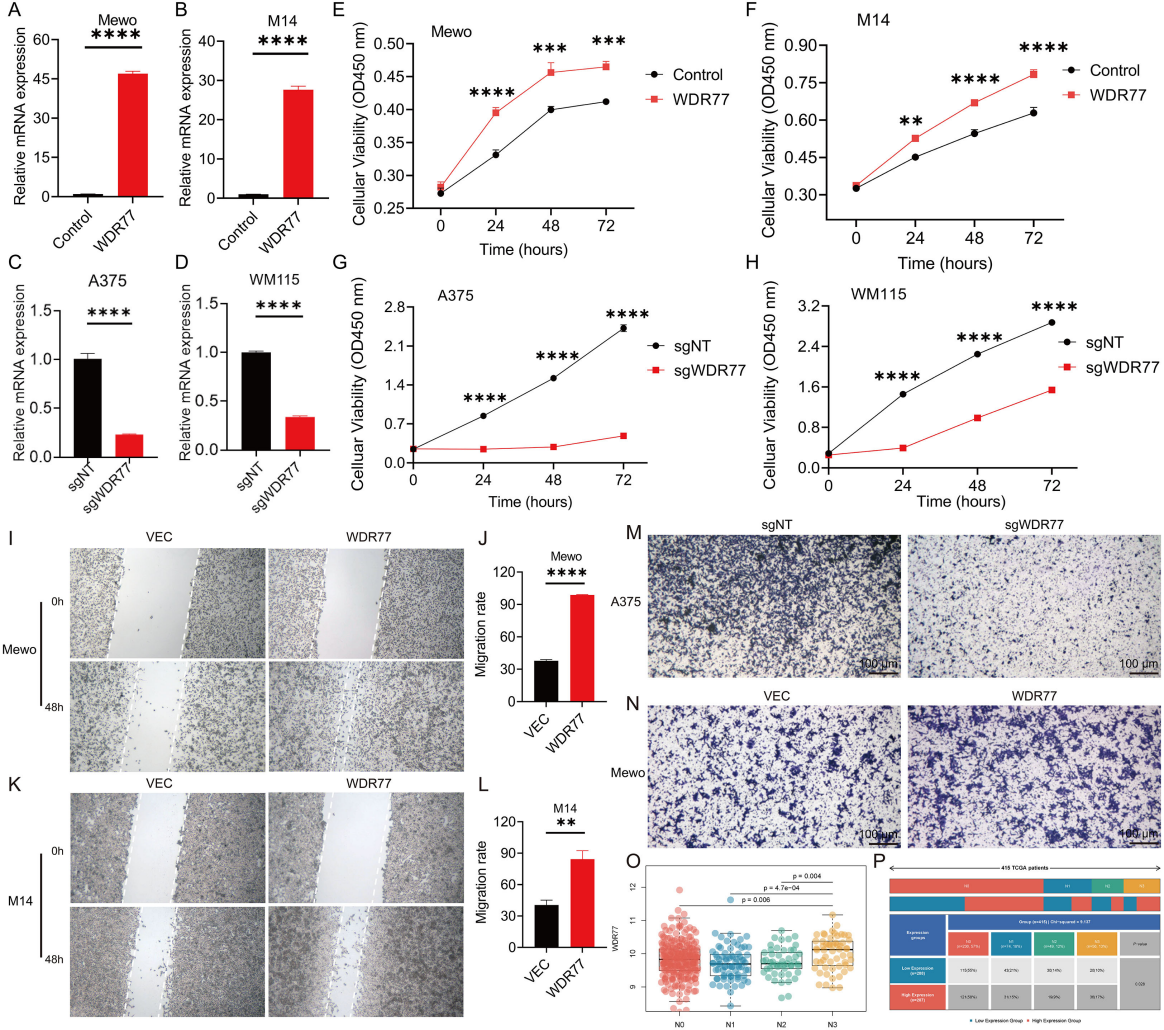
WDR77 promotes melanoma cell proliferation and migration. **(A–D)** qRT-PCR confirms WDR77 overexpression/knockout in Mewo **(A)**, M14 **(B)**, A375 **(C)**, and WM115 **(D)** cells (P < 0.0001). **(E–H)** CCK-8 assays show WDR77 overexpression increases cell viability in Mewo **(E)** and M14 **(F)** cells, while WDR77 knockout decreases cell viability in A375 **(G)** and WM115 **(H)** cells. **(I–L)** Wound healing assays show WDR77 overexpression strongly promotes cell migration in Mewo **(I, J)** and M14 **(K, L)** cells. **(M, N)** Transwell migration assays providing bidirectional validation: WDR77 knockout in A375 **(M)** cells reduces migration, while WDR77 overexpression in Mewo **(N)** cells enhances migration. Scale bars: 100 μm. **(O)** Box plots showing WDR77 expression levels across N staging groups, with highest expression in N3 stage compared to earlier stages (N0 vs N3: p = 0.006; N1 vs N3: p = 4.7e−04; N2 vs N3: p = 0.004). **(P)** Bar plot showing that high WDR77 expression correlates with more advanced N staging (P = 0.028). ** means p < 0.01, *** means p < 0.001, **** means p < 0.0001.

The quantitative reverse transcription polymerase chain reaction (qRT-PCR) analysis confirmed that the overexpression of WDR77 was successfully achieved in Mewo and M14 cells, and the gene knockout was achieved in A375 and WM115 cells (**Figures 6A–D**). CCK-8 assays showed that WDR77 overexpression significantly promotes the cell viability of Mewo and M14 cells (**Figures 6E, F**), whereas WDR77 knockout significantly inhibits the cell viability of A375 and WM115 cells (**Figures 6G, H**).

Besides, wound-healing assays demonstrated that the overexpression of WDR77 significantly accelerated the wound closure of Mewo (**Figures 6I, J**) and M14 cells (**Figures 6K, L**), and the migration rate was significantly higher than that of the control group. The Transwell experiment provided two-way verification, indicating that WDR77 gene knockout reduced the migration of A375 cells, while WDR77 overexpression enhanced the migration of Mewo cells (**Figures 6M, N**).

This migration-promoting effect of WDR77 was further supported by the clinical data of the TCGA-SKCM cohort. Namely, the expression level of WDR77 was significantly higher in the late N3 phase than in the early stage (N0 vs N3: p = 0.006; N1 vs N3: p = 4.7e−04; N2 vs N3: p = 0.004) (**Figure 6O**), and high WDR77 expression was significantly associated with more advanced N staging (P = 0.028) (**Figure 6P**).

**Figure 7:**
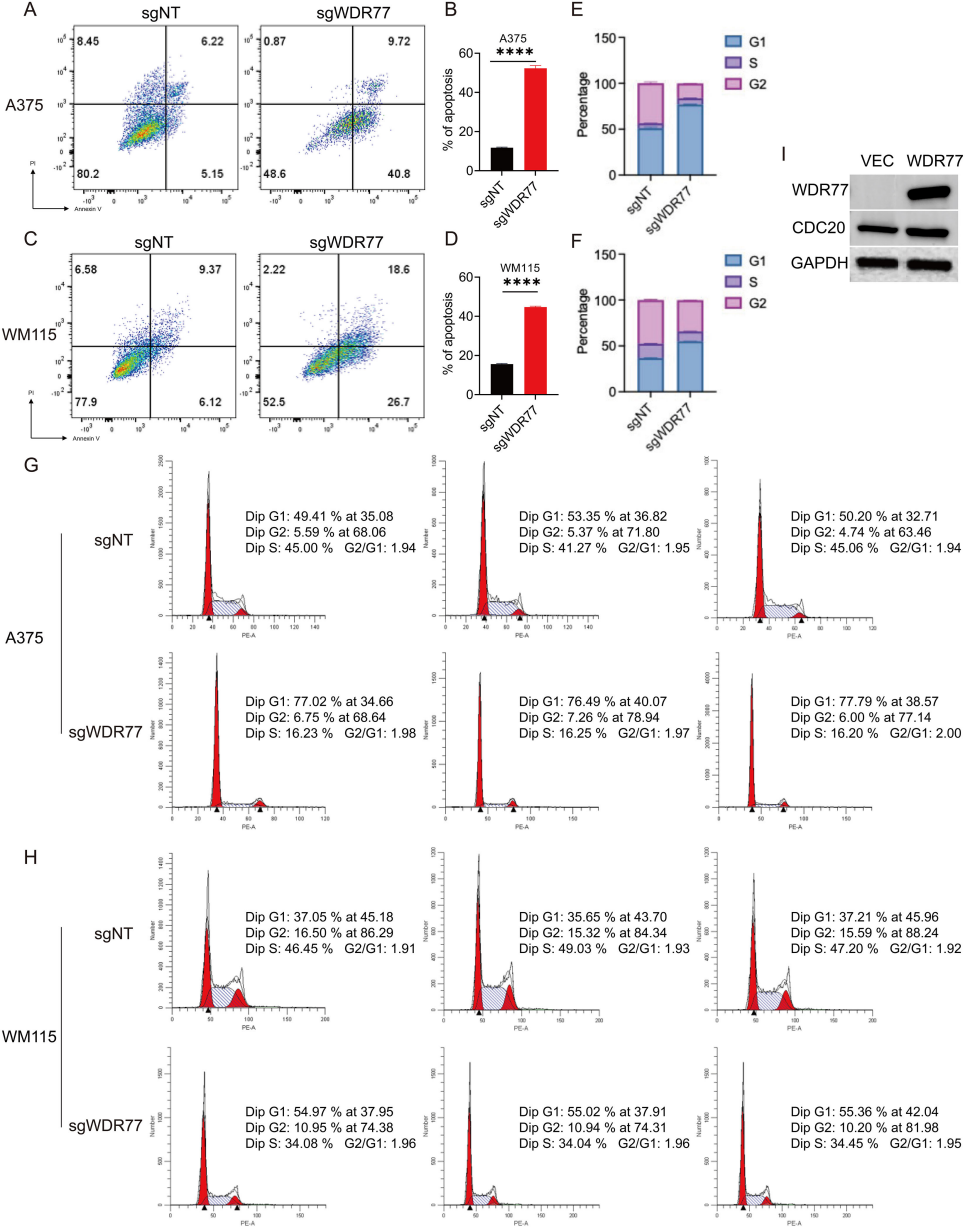
WDR77 regulates cell cycle progression, apoptosis, and CDC20 expression in melanoma cells. **(A–D)** Flow cytometry showing increased apoptosis upon WDR77 knockout in A375 **(A, B)** and WM115 **(C, D)** cells. **(E–H)** Cell cycle analysis demonstrating G1 arrest upon WDR77 knockout in A375 **(E, G)** and WM115 **(F, H)** cells. **(I)** Western blot showing CDC20 upregulation upon WDR77 overexpression in Mewo cells. ****p < 0.0001.

Flow cytometry analysis showed that in A375 cells (**Figures 7A, B**) and WM115 cells (**Figures 7C, D**), the knockout of WDR77 significantly increased apoptosis rate, which was significantly different from the control group. Cell cycle analysis showed that the absence of WDR77 leads to stage G1 stagnation in A375 cells (**Figures 7E, G**) and WM115 cells (**Figures 7F, H**). Western blot analysis confirmed that the overexpression of WDR77 upregulated the expression of CDC20 in Mewo cells (**Figure 7I**).

## Discussion

Skin cutaneous melanoma (SKCM) is a highly aggressive cancer with the potential for rapid progression, which highlights the urgent need for novel prognostic biomarkers. To address this challenging issue, we have developed a histone-related signature for SKCM through systematic integration of 101 machine learning algorithm combinations. The optimal seven-gene model (RSF-SuperPC, C-index = 0.655) grouped patients into different risk subgroups with differential survival trajectories. Random forest analysis identified WDR77 as the most important prognostic gene. After that, we investigated the central role of WDR77 in melanoma pathogenesis.

WD repeat-containing protein 77 (WDR77) (also known as MEP50, p44, or Valois) is an essential cofactor of the protein arginine methyltransferase PRMT5, responsible for regulating methylation of histone and protein, thus regulating chromatin structure, transcription, cell cycle progression, and RNA splicing (39, 40). Although WDR77 has been implicated in various malignancies (41–43), its specific role in the pathogenesis of melanoma is still unclear. Thus, we began by examining its expression, and found that at the level of mRNA and protein, the expression of WDR77 in melanoma was significantly higher than that of normal skin tissue. Patients with the highest WDR77 expression exhibited the worst survival outcomes and the mortality rate was always the highest. Importantly, single-cell analyses demonstrated that WDR77 is commonly expressed in malignant cells of various cancer types, and this pattern was also observed in melanoma-specific datasets. In the Meta-analysis of independent cohorts, it was confirmed that WDR77 is a reliable risk factor for the adverse outcome of SKCM. Beyond that, WDR77 expression increased with disease progression, showing a significant rise in the N3 stage compared to earlier stages. Therefore, we explored the molecular basis of its carcinogenic activity. Immunofluorescence showed that WDR77 is distributed in the nucleus and cytoplasm. To explore this functional role, we verified that overexpression of WDR77 increases cell viability and migration rates, while knocking out WDR77 inhibits cell proliferation and motility. Based on these results, WDR77 has been determined as a key contributor to the invasive phenotype of melanoma.

Next, our analysis strongly linked WDR77 expression to cell cycle dysregulation in melanoma. This function is conserved in other cancers; for instance, in breast cancer, WDR77 drives G1/S phase transition after being stabilized by HNRNPC (44). Furthermore, another cell-cycle related molecule was identified. Cell division cycle 20 (CDC20) is a critical cofactor of the anaphase-promoting complex/cyclosome (APC/C). It can control mitotic progression through timely degradation of cell cycle proteins such as securin and cyclin B1 (45–48). CDC20 overexpression can lead to poor clinical outcomes in multiple cancers (49–51). In melanoma, high CDC20 expression means aggressive disease behavior and metastatic potential (52). CDC20 is also deemed as a therapeutic target, for its inhibitors show efficacy in preclinical melanoma models, including the ability to overcome BRAF inhibitor resistance (53). Also, our analysis results demonstrated positive relations between WDR77 and CDC20 in melanoma before we thoroughly examined their functional relationship in melanoma progression.

WDR77 knockout led to G1 phase accumulation and increased apoptosis in melanoma cells. These two effects together explain the reduced cell viability observed in CCK-8 assays. We then examined DNA replication and found that cells entering S phase showed normal EdU incorporation (**Supplementary Figures 5A, B**), suggesting WDR77 does not affect DNA synthesis itself. Instead, the G1 accumulation indicates that WDR77 primarily acts at the G1/S checkpoint. Without WDR77, most cells cannot exit G1 phase, but those that pass this checkpoint replicate DNA normally. This means WDR77 regulates G1/S transition efficiency rather than DNA replication, consistent with its role in gene expression control. The G1/S specific block also explains why cells accumulate in G1 rather than G2/M phase, since WDR77-depleted cells are arrested before reaching M phase where CDC20 typically functions. Although WDR77 and CDC20 function at different cell cycle stages, their expression patterns are closely linked. WDR77-CDC20 co-high expression strongly associates with the poorest survival in SKCM patients. Additionally, WDR77 overexpression upregulated CDC20 protein levels. These findings suggest WDR77 may regulate CDC20 transcriptionally or post-transcriptionally. Given WDR77’s G1/S-specific phenotype and its role as a PRMT5 cofactor for histone methylation, it likely acts through downstream effectors such as G1/S cyclins or CDK inhibitors (54, 55).

To validate WDR77-CDC20 co-expression at the cellular level, we performed single-cell and spatial transcriptomic analyses. Pan-cancer single-cell data showed that WDR77 and CDC20 are co-expressed, which was confirmed in two melanoma-specific datasets. Spatial transcriptomics further revealed that both genes are co-localized in malignant cell enrichment spots, where their expression is significantly higher than in normal tissue. This spatial pattern supports their coordinated expression during melanoma progression.

Melanoma treatment has improved with immunotherapy, but many patients do not respond or develop resistance. Identifying alternative therapeutic strategies for these patients remains an urgent clinical need. Beyond cell cycle regulation, we found that WDR77 relates to tumor immunity. In melanoma, WDR77 expression negatively correlates with CD8+ T cell recruitment and high WDR77 promoter methylation associated with the improved survival. In COAD and STAD, WDR77 was lower in MSI-high tumors, which typically respond better to immunotherapy. These findings suggest WDR77 may influence the tumor immune microenvironment, though the mechanism requires further validation.

These findings prompted us to explore the therapeutic implications of targeting WDR77. The WDR77-PRMT5 complex plays a recognized carcinogenic role in many cancers (40, 43, 56–58). We observed co-upregulation of WDR77 and PRMT5 in melanoma, suggesting that patients with high WDR77 expression may benefit from PRMT5-targeted therapies currently under clinical evaluation (59, 60). Future studies should verify whether high WDR77 expression can identify patients likely to respond to PRMT5 inhibitors. Importantly, since PRMT5 inhibition enhances checkpoint immunotherapy in melanoma models (17), combining PRMT5 inhibitors with immunotherapy may provide a treatment option for patients who fail immunotherapy alone. This combination strategy warrants further clinical investigation.

In conclusion, this study establishes WDR77 as both a prognostic marker and functional regulator in melanoma. We demonstrated that WDR77 promotes cell proliferation and migration through regulating the G1/S checkpoint, and identified its co-expression with CDC20. However, there are still some important issues that need to be addressed. Future research should validate these findings *in vivo* and clarify their molecular mechanisms in greater detail. Specifically, we need to clarify how WDR77 regulates the G1/S transition, how it cooperates with PRMT5, and whether it directly or indirectly regulates the expression of CDC20. Understanding these mechanisms will be crucial for evaluating treatment strategies. Given that PRMT5 inhibitors are currently in clinical trials and WDR77-CDC20 co-high expression predicts poor outcomes, the PRMT5-WDR77 complex represents a promising therapeutic target. Whether PRMT5 inhibition should be used alone or combined with immunotherapy in melanoma patients, particularly those who fail immunotherapy, requires further clinical investigation.

## Data Availability

The original contributions presented in the study are included in the article/**Supplementary Material**. Further inquiries can be directed to the corresponding authors.

## Supplementary material

Supplementary Figure 1 WDR77 expression in cutaneous squamous cell carcinoma (cSCC), atopic dermatitis (AD), and psoriasis. **(A–C)** Bar charts showing significantly higher WDR77 expression in malignant regions compared to boundary and non-malignant regions in cSCC tissue sections. **(D, E)** Differential expression analysis demonstrating WDR77 upregulation in AD **(D)** and psoriasis **(E)** lesions versus non-lesional skin across multiple independent cohorts.

Supplementary Figure 2 PRMT5 expression in melanoma and its association with WDR77. **(A)** Beeswarm plot showing PRMT5 expression is significantly upregulated in melanoma tissues compared to normal skin tissues using integrated TCGA-SKCM and GTEx data (P = 1.557e-05). **(B)** Raincloud plot demonstrating significant differences in WDR77 expression across PRMT5 expression quartiles (P = 0.02).

Supplementary Figure 3 WDR77 expression shows heterogeneous correlations with immune cell infiltration. Heatmaps showing correlations between WDR77 expression and immune cell infiltration levels. Color indicates correlation coefficient (red: positive; blue: negative). × indicates non-significant correlations (P ≥ 0.05).

Supplementary Figure 4 Association between WDR77 expression and tumor immunity patterns. **(A)** Correlation matrix showing a negative association between WDR77 expression and CD8+ T cell recruitment score. **(B, C)** Box plots demonstrating WDR77 expression levels across microsatellite instability (MSI) subtypes in colon adenocarcinoma (COAD) **(B)** and stomach adenocarcinoma (STAD) **(C)**. MSI-high (MSI-H) tumors exhibit significantly lower WDR77 expression compared to MSI-low (MSI-L) and microsatellite stable (MSS) subtypes. **(D, E)** Heatmaps showing the distribution of patients across WDR77 expression groups (high/low) and MSI subtypes in COAD **(D**) and STAD **(E)**. **(F)** Kaplan-Meier survival curves demonstrating that WDR77 promoter hypermethylation is associated with improved DSS in SKCM (P = 0.026).

Supplementary Figure 5 EdU incorporation assays in WDR77 knockdown melanoma cells. **(A, B)** EdU-positive cell percentages in A375 **(A)** and WM115 **(B)** cells with WDR77 knockout or control. Representative flow cytometry histograms and quantification showing no significant differences.
