# Supplementary figures and images for: Histone-related gene WDR77 promotes tumor progression through cell cycle regulation in skin cutaneous melanoma
Source: Front Immunol. 2025 Dec 4;16:1611112. doi: 10.3389/fimmu.2025.1611112 (PMC12711826; doi:10.3389/fimmu.2025.1611112)

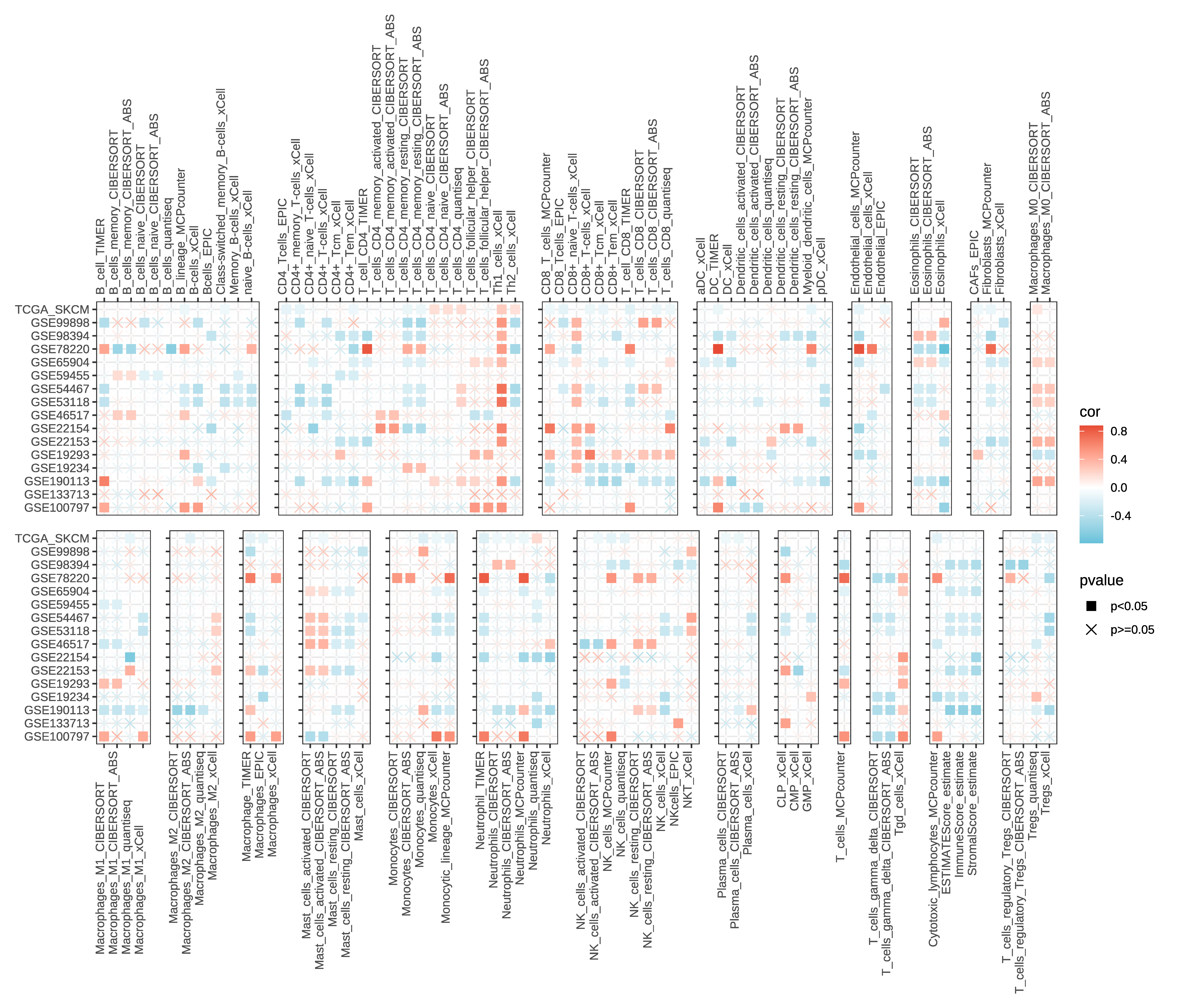

Supplement: Supplementary Figure 1 — WDR77 expression in cutaneous squamous cell carcinoma (cSCC), atopic dermatitis (AD), and psoriasis. (A–C) Bar charts showing significantly higher WDR77 expression in malignant regions compared to boundary and non-malignant regions in cSCC tissue sections. (D, E) Differential expression analysis demonstrating WDR77 upregulation in AD (D) and psoriasis (E) lesions versus non-lesional skin across multiple independent cohorts. [file DataSheet2.zip › Supplementary Fig3.jpg]
